# Changes of Attention during Value-Based Reversal Learning Are Tracked by N2pc and Feedback-Related Negativity

**DOI:** 10.3389/fnhum.2017.00540

**Published:** 2017-11-07

**Authors:** Mariann Oemisch, Marcus R. Watson, Thilo Womelsdorf, Anna Schubö

**Affiliations:** ^1^Department of Biology, Centre for Vision Research, York University, Toronto, ON, Canada; ^2^Department of Psychology, Vanderbilt University, Nashville, TN, United States; ^3^Department of Psychology, Philipps-University Marburg, Marburg, Germany

**Keywords:** visual selective attention, attentional learning, feedback, N2pc, reversal learning, EEG, reward, value learning

## Abstract

Previously learned reward values can have a pronounced impact, behaviorally and neurophysiologically, on the allocation of selective attention. All else constant, stimuli previously associated with a high value gain stronger attentional prioritization than stimuli previously associated with a low value. The N2pc, an ERP component indicative of attentional target selection, has been shown to reflect aspects of this prioritization, by changes of mean amplitudes closely corresponding to selective enhancement of high value target processing and suppression of high value distractor processing. What has remained unclear so far is whether the N2pc also reflects the flexible and repeated behavioral adjustments needed in a volatile task environment, in which the values of stimuli are reversed often and unannounced. Using a value-based reversal learning task, we found evidence that the N2pc amplitude flexibly and reversibly tracks value-based choices during the learning of reward associated stimulus colors. Specifically, successful learning of current value-contingencies was associated with reduced N2pc amplitudes, and this effect was more apparent for distractor processing, compared with target processing. In addition, following a value reversal the feedback related negativity(FRN), an ERP component that reflects feedback processing, was amplified and co-occurred with increased N2pc amplitudes in trials following low-value feedback. Importantly, participants that showed the greatest adjustment in N2pc amplitudes based on feedback were also the most efficient learners. These results allow further insight into how changes in attentional prioritization in an uncertain and volatile environment support flexible adjustments of behavior.

## Introduction

Visual selective attention allows the prioritization of task-relevant over irrelevant stimuli in the visual field. Traditionally, selective attention has been dissociated into goal-directed “top-down” driven selective attention and salience-driven “bottom-up” selective attention (e.g., Posner and Petersen, 1990; Kastner and Ungerleider, 2000; Corbetta and Shulman, 2002). However, in recent years it has become evident that this dichotomy does not suffice to explain all instances in which a stimulus, or a set of stimuli, become the target of attentional priority (Awh et al., 2012; Anderson, 2013; Womelsdorf and Everling, 2015). A third source of attentional control, referred to as “experience-driven”, includes an individual’s recent selection-history and reward learning (Della Libera and Chelazzi, 2006; Anderson et al., 2011a; Awh et al., 2012; Irons and Leber, 2016). In particular, previously learned reward value has been shown to be a strong modulator of attentional prioritization (e.g., Della Libera and Chelazzi, 2009; Krebs et al., 2010; Anderson et al., 2011b, 2013, 2014; Della Libera et al., 2011; Hickey et al., 2011, 2015; Sali et al., 2014; Bucker and Theeuwes, 2017). For example, non-salient and task-irrelevant distractors that have previously been associated with reward can capture attention involuntarily and cause slower reaction times (RTs) in classical visual search tasks, and this is modulated by reward level, such that the higher the previously-associated reward, the greater the subsequent capture (e.g., Anderson et al., 2011b, 2013; Munneke et al., 2015).

Neurophysiological evidence for this increased attentional capture by high-valued stimuli has been found in the mean amplitude of the N2pc (e.g., Kiss et al., 2009; Feldmann-Wüstefeld et al., 2015, 2016; Sawaki et al., 2015). The N2pc is an EEG component thought to reflect attentional target selection processes (Luck and Hillyard, 1994a; Woodman and Luck, 2003; Eimer and Grubert, 2014; Eimer, 2014), likely generated in intermediate and high levels of the ventral visual processing pathway (Hopf et al., 2000, 2006). It onsets earlier and is more pronounced when search targets are associated with a higher value (Kiss et al., 2009), and the presence of higher value distractors causes a decrease in target enhancement and an increase in distractor suppression during visual search (Feldmann-Wüstefeld et al., 2016). Sawaki et al. (2015) found that prior to visual search, a high value cue elicited stronger distractor suppression than a low value cue, and thereafter a smaller N2pc component was observed during the visual search. The authors argue that increased suppression to a high value but non-informative (to target selection) cue may have allowed better performance in the following visual search, which was supported by decreased RTs as well as decreased alpha oscillation levels prior to the visual search that indicated heightened visual readiness (Sawaki et al., 2015).

We have thus already gained substantial insight into the behavior and neurophysiological processes that underlie the selective processing of high- or low-valued target and distractor signals. However, the distinction between targets and distractors is often much less clear outside the classic experimental environment. Real life is substantially more volatile, and therefore the stimuli that surround us must continuously be reevaluated with regards to their relevance to our current goals. A critical goal of attentional prioritization is likely reward maximization and loss minimization (e.g., Navalpakkam et al., 2010), meaning that in a dynamic world we have to continuously learn and update our choice criteria with regards to the stimuli we attend.

We do not yet know how flexibly attentional target selection, as tracked by the N2pc component, can change in response to changes in reward values. In this study, we therefore employed a value-based reversal learning task in which stimulus reward values changed repeatedly and without warning. Specifically, participants were asked to attend to and choose one of two target stimuli that differed in color and likelihood of leading to a high reward outcome. This color-value association changed often and unannounced, such that the previously high value stimulus became the low value stimulus and vice versa. Participants therefore had to continuously re-evaluate, based on trial and error, whether they were choosing the currently high valued stimulus. This allowed us to assess learning-related changes in behavior, and to compare neural processing when subjects were in the process of learning the current value contingency, with processing when they had already successfully learned the current value contingency. Using EEG recordings, we examined learning- and choice-related differences to the N2pc. Since participants used trial-by-trial feedback to evaluate choices, we also examined feedback-related differences to the N2pc and learning-related differences to frontal feedback related negativity (FRN), which has previously been shown to change during reversal learning and has been suggested to encode prediction error signals and behavioral adjustments (e.g., Cohen and Ranganath, 2007; Chase et al., 2011; Walsh and Anderson, 2011).

**Figure 1:**
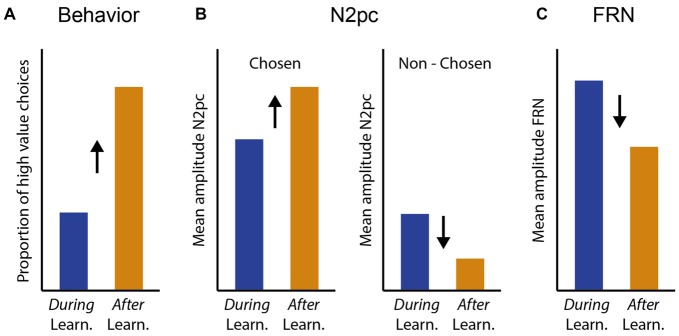
Hypotheses for learning-related changes in behavior, N2pc and feedback related negativity (FRN) amplitudes. **(A)** Successful learning (*after* learning) in our value based reversal learning task is reflected in an increased probability of choosing the currently high valued target. **(B,C)** We expected N2pc and FRN amplitudes to change in parallel with learning behavior. We hypothesized that N2pc amplitudes for the stimulus that was chosen for response, and therefore presumably actively attended, would increase with learning, to potentially reflect more accurate attentional target selection with successful learning (**B**, left). In contrast, we hypothesized that N2pc amplitudes for the stimulus that was *not* chosen for response, and therefore presumably *not* actively attended, would be substantially smaller than that for the stimulus chosen for response, and would further decrease with learning, to potentially reflect more successful avoidance of attentional capture by the distracting stimulus (**B**, right). **(C)** We hypothesized that FRN amplitudes would be greater *during* learning of the current value contingency compared with *after* learning, potentially reflecting the greater propensity to actively assess feedback during periods that may require behavioral adjustment.

Neural processing of the valued stimuli was isolated by always placing one valued stimulus on the vertical midline, thereby attributing the lateralized EEG activity to the second, lateralized stimulus (e.g., Hickey et al., 2009; Feldmann-Wüstefeld et al., 2016). Importantly, our task design did not have a fixed dissociation into “target” and “distractor”, since either of the two stimuli could be selected for response and the identity of the high and low value stimuli changed frequently. Instead, we differentiated processing of the selected (target) and the non-selected (distractor) stimulus on a trial-by-trial basis dependent on participants’ choice performance. We expected to observe learning-dependent changes in attentional prioritization reflected in N2pc amplitudes, and in feedback-processing reflected in FRN amplitudes (Figure 1). When performing similar tasks (e.g., Cools et al., 2002; Chase et al., 2011) participants generally show a low probability of choosing the newly high-valued stimulus in the trials immediately following a value reversal and therefore *during* learning of the current value contingency, and a high probability of choosing the currently high valued stimulus *after* successful learning (Figure 1A). We hypothesized that changes in N2pc and FRN amplitudes would parallel these changes in learning behavior. Specifically, we hypothesized that with successful learning of the current value contingency, the N2pc elicited by the stimulus selected for response (and therefore presumably actively attended), should increase, potentially reflecting more precise attentional selection of the current target stimulus (Figure 1B, left). We furthermore hypothesized that the N2pc elicited by the stimulus that was *not* selected for response (therefore presumably not actively attended) should generally be smaller than that of the stimulus that was selected for response, and it should further decrease with successful learning, potentially reflecting more successful avoidance of attentional capture by the current distractor stimulus (Figure 1B, right). Alternatively, it is possible that relatively fast learning during frequent value reversals is not reflected in changes in early attentional stimulus selection as measured with the N2pc, or that attentional processing of only the current target or only the current distractor is affected. Finally, we expected feedback processing reflected in FRN amplitudes to be greater *during* learning of the current value contingency compared with *after* learning, potentially reflecting the greater propensity to keep track of accumulated feedback when behavior needs to potentially be adjusted following a value reversal (Figure 1C, Chase et al., 2011).

## Materials and Methods

### Participants

Twenty-six students of the Philipps-University Marburg participated in the experiment for payment (8€/h) or course credit. Contingent on performance, participants could collect an additional monetary bonus of up to 6€. Three participants were rejected from analysis because too many trials (>40%) were lost due to EEG artifacts and non-learning (see “Data Analysis” section). Two participants had to be rejected due to technical issues. Analyses are shown for the remaining 21 participants (15 females, 6 males, mean age = 21.4). Three out of those 21 participants were left-handed. All participants were naïve to the purpose of the experiment and had normal or corrected-to-normal visual acuity and normal color vision. Visual acuity and color vision were tested with an OCULUS Binoptometer 3 (OCULUS Optikgeräte GmbH, Wetzlar, Germany). This study was carried out in accordance with the recommendations of the Ethics Committee of the Department of Psychology at the Philipps University Marburg with written informed consent from all participants, in accordance with the Declaration of Helsinki.

### Apparatus and Stimuli

**Figure 2:**
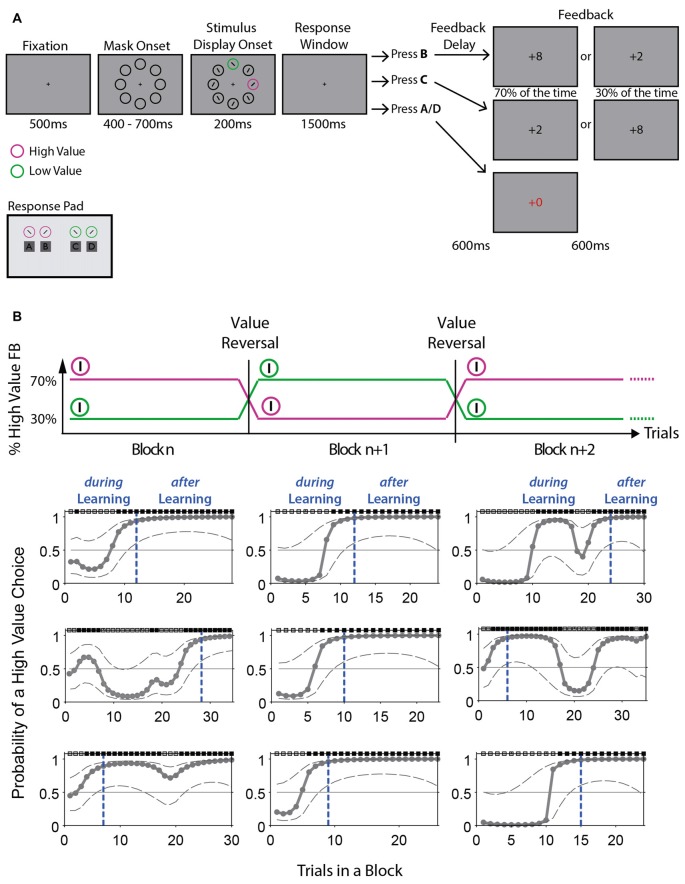
Value-based reversal learning task and example block performance computed with Expectation Maximization (EM) algorithm. **(A)** The task started with a central fixation cross, followed by the appearance of eight black circles. For the target display, lines with different orientation appeared in all circles and two circles changed color to pink and green (or blue and orange). Participants had 1500 ms to report the line orientation (+45° or −45° tilt) inside one of the two colored stimuli. Selecting the high value stimulus would lead to a high value feedback (+8) in 70% of the trials and to a low value feedback (+2) in 30% of the trials. This was reversed for the low value stimulus. Feedback was presented for 600 ms. If an incorrect color-line orientation combination was reported, a +0 was shown in red font for 1200 ms. After 1000–1300 ms, a new trial was initiated. The response pad used by participants is illustrated below the task. **(B)** Top: schematic illustration of the value reversals applied to the colored stimuli in consecutive blocks. Bottom: displayed is the probability of a high value choice across trials for 9 example blocks performed by one representative participant. The probability of a high value choice was computed using an EM algorithm (see “Materials and Methods” section and Smith et al., 2004). Dotted gray lines represent 95% confidence intervals. Dotted blue lines indicate the trial at which learning has occurred (T_Learn_) according to the ideal observer confidence interval. Trials are split into *during* learning and *after* learning trials according to T_Learn_. Black and gray boxes above plots indicate high value and low value choice trials, respectively.

Participants were seated in a comfortable chair in a dimly lit and electrically shielded room, facing a monitor placed at a distance of approximately 100 cm from their eyes. Stimuli were presented on a 22″ screen (1680 × 1050 px) using Unity3D 5.3.5 (Unity Technologies, San Francisco, CA, USA). The display showed eight stimuli (diameter of 2.7°) arranged equidistantly on an imaginary circle with an eccentricity of 5.5° of visual angle (Figure 2A). All stimuli were presented against a gray background. Six of the stimuli were dark gray circles (RGB 24, 24, 24); the remaining two stimuli were one of two different target colors. Half the participants were presented with one pink (RGB 237, 83, 255) and one green (RGB 29, 181, 13) circle, half with one orange (RGB 217, 148, 14) and one blue (RGB 44, 168, 255) circle. All four possible stimulus colors were approximately isoluminant with the gray background (45.8–55.65 cd/m^2^, luminance background: 51.12 cd/m^2^). Each stimulus contained a black line. Lines inside black stimuli were tilted 30° to the left or the right, alternating around the circle. Lines inside colored target stimuli were always tilted in opposite directions, by 45° to the left or 45° to the right. The two colored stimuli were always separated by one dark gray stimulus, such that one stimulus was always presented on the vertical midline either below or above the fixation cross, while the other was presented laterally to the left or right of the fixation cross. This experimental design was chosen because it allows isolating the processing related to the color stimulus presented laterally from processing of the color stimulus presented vertically. Traditionally, this design is used to isolate target-related from distractor-related processing (e.g., Hickey et al., 2009). However, we do not have a pre-defined target and distractor, rather the same color stimulus changes roles several times throughout the experiment and participants are free to decide which color stimulus they select in a trial (Irons and Leber, 2016; see “Procedure” section below). Thus, we are interested in how observers process stimuli which are associated with different reward values that change roles throughout the experiment.

Four response buttons were arranged on an Ergodex DX1 response pad, such that participants could comfortably place the middle and index fingers of both hands on the buttons. Each participant was randomly assigned to respond to a given stimulus color with the left or right hand, e.g., participants with green and pink stimuli were randomly assigned to respond to pink stimuli with their right hand and green stimuli with their left hand, or vice versa. In either case, the left-most finger of each hand (middle finger left hand and index finger right hand) was used to respond to a 45° tilt to the left (assuming a vertical line) and the right-most finger of each hand (index finger left hand and middle finger right hand) was used to respond to a 45° tilt to the right.

### Procedure

The task is illustrated in Figure 2. Participants were instructed to keep their eyes on the center of the screen throughout a trial. Each trial started with the appearance of a central fixation cross for 500 ms, followed by eight dark gray circles without lines, presented for 400–700 ms. Two circles, one on the vertical midline and one on the horizontal midline, then changed colors, and the lines appeared in all stimuli. Since lines inside colored stimuli were always tilted in opposite directions and participants were free to respond to either color stimulus, two “correct” (high and low value) choices were possible in any given trial (e.g., pink—rightward orientation and green—leftward orientation; trial example from Figure 2A). An incorrect response was recorded when a color/line-orientation pair was reported that was not presented in the display (e.g., pink—leftward orientation and green—rightward orientation). This stimulus display was presented for 200 ms, after which participants had 1500 ms to respond, followed by a delay of 600 ms after which feedback was shown for another 600 ms. Feedback had three possible values: a high-value “+8”, a low-value “+2”, or a “+0” for incorrect responses, the latter shown in red font for 1200 ms. If no response was made within 1500 ms, participants were asked to respond faster in the next trial via a written visual display. The inter-trial interval was 1000–1300 ms.

Each stimulus display always contained the two colored stimuli (e.g., pink and green) and participants freely chose to report the line orientation of either of the two stimuli. At a given time, one color stimulus (e.g., pink) was associated with a 70% probability (high value) of leading to the outcome “+8” and a 30% probability of leading to the outcome “+2”. The second color stimulus (e.g., green) was simultaneously associated with a 30% probability (low value) of leading to an outcome of “+8” and a 70% probability of leading to an outcome of “+2”. Across trials of a block, the color-outcome probability association remained constant for 25 to a maximum of 50 trials (e.g., color 1 high valued). After trial 25, a running average of 80% high value choices over the last 12 trials triggered a block change (e.g., now color 2 high valued), and if this did not occur by trial 50, the block change happened automatically. The reversal was unannounced, requiring the participant to use performance feedback to detect reversals.

Participants were instructed to collect as many points as possible and to respond as fast as possible without jeopardizing response accuracy. They were explicitly informed of the 70%–30% reward outcome distribution and understood that they should optimally always try to choose the color stimulus with the 70% high value outcome probability. Participants were also informed that the color-value associations would change within the experiment. Participants performed a total of 1200 trials, where stimulus positions and target line orientations were pseudo-randomly chosen on each trial. The color that was first associated with a high value was randomly chosen in each experimental session. Each experimental session (1200 trials) lasted approximately 100 min including a 10-min break after 50–60 min. Each participant took part in one experimental session. After the experiment, participants filled in a questionnaire to assess strategies and other factors that may have influenced performance.

### EEG Recording

The EEG was recorded continuously using BrainAmp amplifiers (Brain Products, Munich, Germany) from 64 Ag/AgCl electrodes (actiCAP) positioned according to the international modified 10-20 system. Vertical (vEOG) and horizontal electrooculograms (hEOG) were recorded as voltage difference between electrodes positioned above and below the left eye, and to the left and right canthi of the eyes, respectively. All channels were initially referenced to FCz and re-referenced offline to the average of all electrodes. Electrode impedances were kept below 5 kΩ. The sampling rate was 1000 Hz with a high cut-off filter of 250 Hz (half-amplitude cut-off, 30 dB/oct) and a low cut-off filter of 0.016 Hz (half-amplitude cut-off, 6 dB/oct).

### Data Analysis

Analysis was performed with custom MATLAB code (Mathworks, Natick, MA, USA), utilizing functions from the open-source Fieldtrip toolbox^1^.

#### Behavioral Data

**Figure 3:**
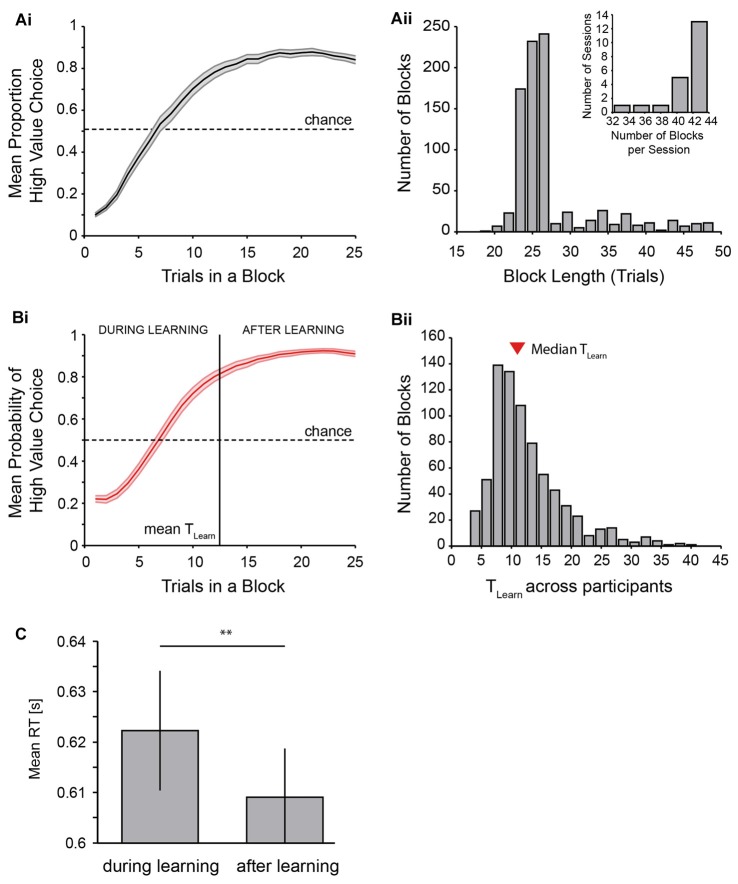
Behavioral performance of the reversal learning task. **(A)** shows performance across all participants with the mean proportion of high value choices across trials **(Ai)** and a histogram of the length of blocks **(Aii)** as well as numbers of blocks per session across all participants (**Aii**, inset). Length of blocks can occasionally be shorter than the minimum of 25 trials because incorrect or late responses were not counted towards block lengths. **(B)** shows performance as measured using the EM algorithm. The mean probability of a high value choice with the average trial at which learning has occurred across participants is shown in **(Bi)**, and the distribution of learning trials across all blocks of all participants is shown in **(Bii)** (mean learning trial: 12.51; median learning trial: 11). **(C)** displays the effect of learning on reaction time (RT) across participants. Two asterisks indicate *p* < 0.01 (*t*-test on RT difference (*during*-*after* learning) across participants).

Incorrect choices, defined as the reporting of a color/line-orientation pair not present in the display (see “Procedure” section), were discarded from all further analyses (5.3 ± 0.12%). To identify at which trial during a block a participant showed statistically reliable learning of the current value rule, we analyzed the trial-by-trial choice dynamics using the state-space framework introduced by Smith and Brown (2003), and implemented by Smith et al. (2004). This framework entails a state equation that describes the internal learning process as a hidden Markov or latent process and is updated with each trial. The learning state process estimates the probability of a high value choice in each trial and thus provides the learning curve of participants (Figures 2B, 3B). The algorithm estimates learning from the perspective of an ideal observer that takes into account all trial outcomes of participants’ choices in a block of trials to estimate the probability that the outcome in a single trial is a high value response or a low value response. This probability is then used to calculate the confidence range of observing a high value response. We defined the learning trial (T_Learn_) as the earliest trial in a block at which the lower confidence bound of the probability for a high value response exceeded the *p* = 0.5 chance level. This corresponds to a 0.95 confidence level for an ideal observer to identify learning. The very first block of an experimental session and blocks in which no learning was identified were removed from further analyses. For most analyses, trials were split based on their occurrence prior to T_Learn_ and after, into *during* learning trials and *after* learning trials, respectively.

RTs *during* and *after* learning were compared by computing the difference between *during* and *after* learning trials per participant and comparing these differences across participants against a distribution with a mean of zero (*t*-test, *α* = 0.05). We tested whether RT and the probability to switch between colors were dependent on the previous trials’ feedback. For the first analysis, we simply compared RT in trial n for trials in which trial n-1 ended with a high value feedback and trials in which trial n-1 ended with a low value feedback. This comparison was made within participants as well as between participants (*t*-test, *α* = 0.05). For the latter analysis, we extracted trials in which a switch of choice was made from stimulus 1 (color 1) to stimulus 2 (color 2) or vice versa. We then computed a ratio of switch trials that followed a low value feedback vs. switch trials that followed a high value feedback. This ratio was then compared across participants against a distribution with a mean of one (*t*-test, *α* = 0.05).

#### EEG Data

For the N2pc analysis the EEG data was segmented into epochs of 700 ms, starting 200 ms prior to stimulus display onset and ending 500 ms after stimulus display onset. The time period from −200 ms to 0 ms was used as baseline. Trials with blinks (vEOG >80 μV) or horizontal eye movements (hEOG >40 μV) were excluded from all analyses (across participants: 78 ± 17 trials). The total trial number available for analysis following artifact removal and removal of non-learned blocks (see “Materials and Methods” section above) was 1047 ± 25 trials across participants. The N2pc was measured at parieto-occipital electrode sites (PO3/4, PO7/8) as lateralized response to the laterally presented colored stimulus. The choice of electrode locations was based on the previously shown topography of N2pc subcomponents (Hickey et al., 2009) and equivalent to earlier studies (Feldmann-Wüstefeld and Schubö, 2013; Feldmann-Wüstefeld et al., 2015). Difference waves were calculated by subtracting activity ipsilateral from activity contralateral to the lateral stimulus, and averaged separately for chosen and non-chosen stimuli to isolate choice-related N2pc differences *during* and *after* learning. In line with previous studies, mean amplitudes for the N2pc were computed for the time interval from 200 ms to 300 ms after stimulus display onset (Luck and Hillyard, 1994b; Woodman and Luck, 1999; Kiss et al., 2008; Hickey et al., 2010). Initial comparisons were made using a two-way repeated-measures analysis of variance (ANOVA) with the factors selection (chosen vs. non-chosen) and learning (during vs. after), and followed up by one-way repeated-measures ANOVAs (*α* = 0.05) with the factor learning conducted separately for chosen and non-chosen stimuli.

To investigate whether feedback in trial n-1 had an impact on the mean amplitude of the N2pc component in trial n, we isolated trials in which a choice to a lateralized color target followed a choice to the same color target presented lateralized, to verify that any effect was solely due to feedback. Specifically, a trial combination (trial n and n-1) was only selected for analysis if, e.g., a response was made to color 1 in trial n-1 and in trial n, and stimulus color 1 was presented at a lateralized position (left or right) in both trials. Following this restriction, total trial numbers available for this analysis across participants were 212 ± 7. These trials were then sorted based on the feedback (high or low) received in trial n-1 and the mean amplitude of the N2pc component in trial n was compared in these two groups of trials. Comparisons were made using two-way repeated-measures ANOVA with the factors feedback value in trial n-1 and learning.

For FRN component analyses we extracted the data into 800 ms epochs, lasting from −200 ms to 600 ms around the feedback event. Similar to previous studies (e.g., Hajcak et al., 2006; Cohen et al., 2007), the FRN component was isolated at the Fz electrode, and as a control additionally at the FCz electrode (see “Results” section, data not shown). Difference waves were calculated by subtracting activity for high value feedback from activity for low value feedback in the 250–325 ms following feedback onset, which generally fell within the time range investigated in previous studies (for review see Walsh and Anderson, 2012). The comparison of FRN mean amplitude *during* vs. *after* learning was computed using two-way repeated measures ANOVA, with the factors feedback value and learning.

To assess a more general effect of learning on feedback processing independent of valence (FRN), we performed a three-way ANOVA with the factors learning, feedback value and time window (12 non-overlapping 50-ms windows ranging from 0 ms to 600 ms post feedback). Follow-up tests of simple main effects were done using one-way ANOVA’s in each time window with *p*-values corrected for multiple comparisons using the Bonferroni-Holm method.

**Figure 4:**
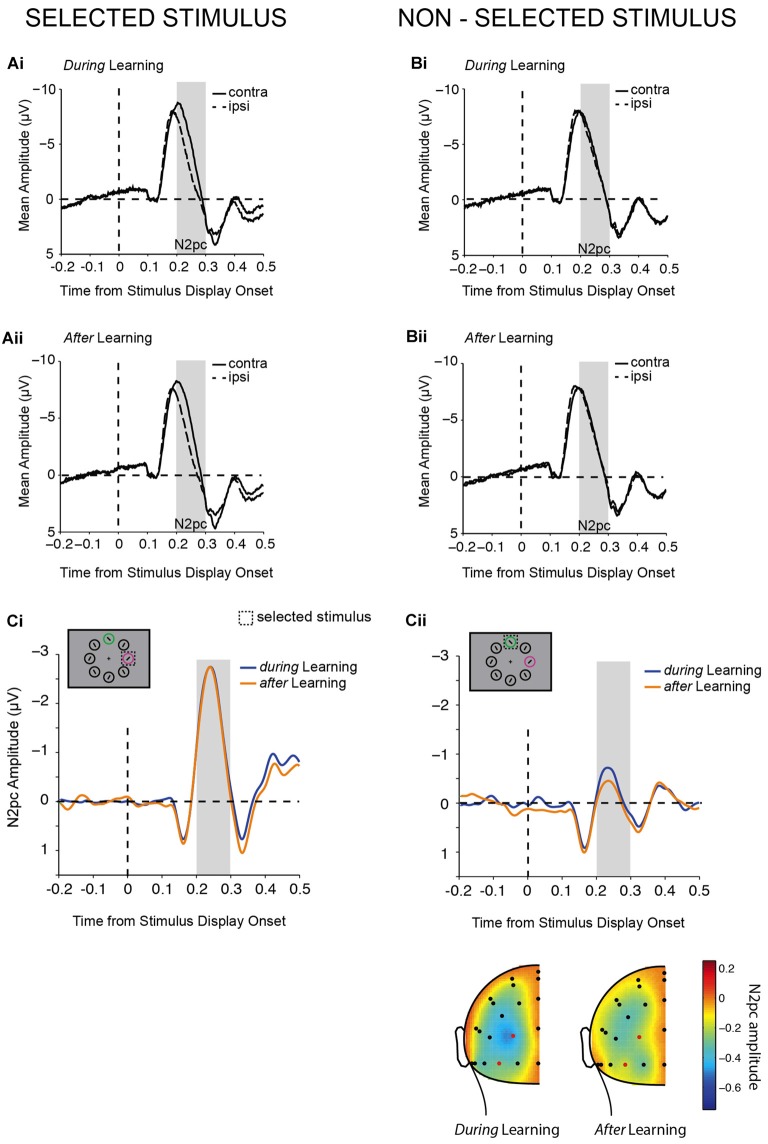
Lateralized N2pc components. **(A,B)** Contra- and ipsi-lateral mean amplitudes from pooled left (PO3, PO7) and right (PO4, PO8) parieto-occipital electrodes are shown aligned to target display onset for lateralized chosen **(A)** and non-chosen **(B)** stimuli *during* **(Ai,Bi)** and *after* **(Aii,Bii)** learning. The gray bars highlight the N2pc time window of analysis (200–300 ms). N2pc difference waves contrasted *during* **(Ci)** and *after* **(Cii)** learning. Example trials are illustrated in the top left corners. The topography of the N2pc (200–300 ms) for non-chosen stimuli is shown below **(Cii)**.

**Figure 5:**
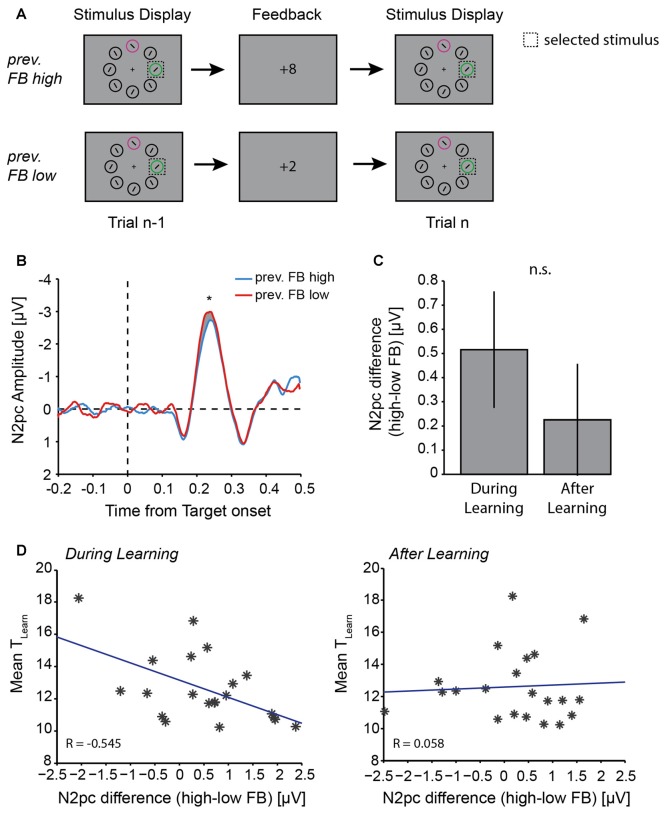
Effect of previous trial feedback on N2pc amplitude. **(A)** Illustration of example trial sequences for “previous feedback high” and “previous feedback low” trials. Trials for analysis were chosen based on the previous trial feedback (n-1), the N2pc analysis was done on trial n. **(B)** N2pc difference wave in trial n following high value (blue line) or low value (red line) feedback in the previous trial. Gray shaded area highlight the N2pc analysis window (200–300 ms after stimulus display onset). Asterisk indicates *p* ≤ 0.05 (one-way ANOVA). **(C)** Mean difference in N2pc amplitude following high vs. low value feedback *during* learning and *after* learning. **(D)** Correlation between individual participants’ T_Learn_ and N2pc amplitude differences between feedback (previous high-value feedback − previous low-value feedback) *during* learning (left) and *after* learning (right). Blue lines represent least square fit. Note that large positive differences indicate a large N2pc difference for high vs. low-value feedback in trial n-1.

**Figure 6:**
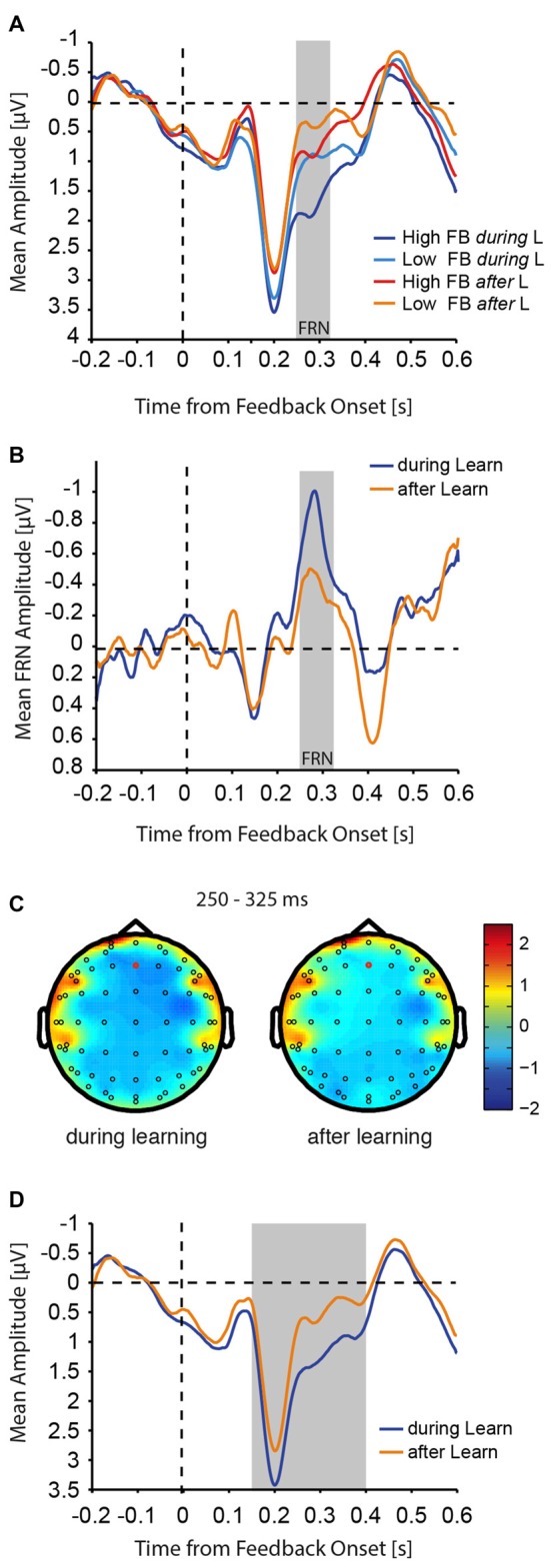
FRN amplitudes. **(A)** Aligned to feedback presentation onset, mean activity at Fz electrode is shown for high and low value feedback *during* and *after* learning trials. **(B)** The mean difference (low value feedback − high value feedback) for the FRN wave is shown *during* and *after* learning. The gray bar indicates the analysis time window for the FRN (250–325 ms after feedback onset). For visualization purposes only, the FRN wave was smoothed with a moving average filter of 5 ms. **(C)** Topography of the FRN (difference elicited by low vs. high value feedback) *during* and *after* learning. Red circles identify Fz electrodes. **(D)** Mean activity from **(A)** is shown collapsed across feedback valence to illustrate the time span of the simple main effect of learning on feedback processing (gray bar).

For visualization purposes only, the N2pc and FRN displayed in Figures 4–6 were smoothed with a moving average filter of 25 ms (40 Hz).

#### Correlations

We compared mean differences in N2pc amplitudes following low vs. high value feedback with mean behavioral measures on an individual participant level using Pearson correlation (*α* = 0.05). The three behavioral measures tested included: (i) the proportion of blocks successfully learned; (ii) the mean trial at which learning was deemed successful across blocks (i.e., T_Learn_); and (iii) mean RT. These three behavioral measures were not correlated across participants (Pearson correlation, all *p* > 0.05). We compared correlation coefficients obtained e.g., *during* learning vs. *after* learning by using a *z*-test to assess differences between two dependent correlations (Steiger, 1980). When the observed *z*-value was greater than |1.96|, we considered the correlation coefficients significantly different.

## Results

### Reversal Learning

Behavioral results are plotted in Figure 3. Participants performed the task very well and generally showed quick increases in the proportion of high value choices following a value reversal (Figures 2B, 3Ai,Bi), in line with the behavioral assumptions (Figure 1A). This is further shown in the distribution of block lengths observed across all participants, whereby the majority of blocks had a length of approximately 25 trials, indicating a performance of 80% high value choices around the time trial 25 was reached (Figure 3Aii, note that blocks shorter than 25 trials were possible following the rejection of incorrect responses, see “Materials and Methods” section). Participants performed a mean of 41.1 blocks per experimental session. 86% out of all blocks were successfully learned (across participants: 86 ± 1.6%). Learning of the current value rule across blocks and participants occurred within a mean of 12.5 ± 0.5 trials as identified using the Expectation Maximization algorithm by Smith et al. (2004; Figure 3B, median: 11).

RT significantly decreased with learning of the current value rule. Participants showed on average 13.2 ms shorter RTs in trials after acquiring the current rule (*after* learning, 609 ± 10 ms) as opposed to trials beforehand (*during* learning, 622 ± 12 ms; *t*_(20)_ = 3.31, *p* = 0.004, Figure 3C).

### Attention Deployment Changes with Learning

Mean amplitudes for the N2pc were computed for the time interval from 200 ms to 300 ms after stimulus display onset (Figure 4). An initial two-way repeated-measures ANOVA tested the effects of the factors selection and learning on N2pc amplitudes. A main effect of selection (*F*_(1,20)_ = 44.04, *p* < 0.001) showed that a pronounced N2pc was elicited when the chosen stimulus was presented at a lateralized position (Δ_(contra-ipsi)_ = −1.901 ± 0.29 μV), which was substantially reduced when the non-chosen stimulus was presented laterally and the chosen stimulus on the vertical midline (Δ_(contra-ipsi)_ = −0.271 ± 0.16 μV). A main effect of learning additionally suggested that N2pc amplitudes differed *during* learning and *after* learning (*F*_(1,20)_ = 10.79, *p* = 0.004). The *post hoc* comparison showed that N2pc amplitudes were significantly larger (more negative) *during* learning (Δ_(contra-ipsi)_ = −1.155 ± 0.20 μV) than *after* learning (Δ_(contra-ipsi)_ = −1.016 ± 0.20 μV). Therefore, as initially predicted (Figure 1B) we found main effects of both learning and selection on the N2pc. However, contrary to our expectation (Figure 1B), we did not find a significant interaction between the two (*F*_(1,20)_ = 1.04, *p* = 0.319). While the absolute magnitude of the N2pc for the non-chosen stimulus was higher *during* learning than *after* learning (Figure 4Cii), and this was significant as a main effect of a one-way ANOVA (*F*_(1,20)_ = 5.36, *p* = 0.031) as predicted (Figure 1B, right), the magnitude for the chosen stimulus was virtually identical *during* and *after* learning (*F*_(1,20)_ = 1.41, *p* = 0.249, Figure 4Ci), which does not match our prediction (Figure 1B, left). Thus, our results suggest that the primary effect of learning on the N2pc amplitude in our task is to suppress attention to non-chosen distractors, rather than to enhance attention to chosen targets. The lack of apparent target enhancement might explain why the two-way interaction was not significant, despite the apparent effect of learning on the non-chosen stimulus. Given this lack of a significant interaction, the differential results should be treated as suggestive, rather than definitive.

In summary, N2pc results showed that attention was mainly deployed to the chosen stimulus compared with the non-chosen stimulus, and that attention deployment was generally more pronounced *during* learning compared with *after* learning. In contrast to our hypotheses (Figure 1B), the direction of the effects of learning were not opposing for chosen and non-chosen stimuli. Thus, even though we did not observe an interaction between learning and selection, we nevertheless found evidence suggestive of successful learning mainly leading to a decrease in processing of the non-chosen compared with the chosen stimulus (Figure 4C). We therefore found partial evidence in line with our hypothesis for the effect of learning on non-chosen stimuli (Figure 1B, right).

### Low Value Feedback Is Followed by Increased Attentional Target Selection

To investigate the impact of low or high value feedback on behavioral or electrophysiological measures, we assessed whether RT, probability to switch color choices, and N2pc mean amplitudes were affected by the previous trial’s feedback. Whether the feedback in trial n-1 was of high or low value had no impact on RT in the following trial n (across all trials: *t*_(12,470)_ = −0.36, *p* = 0.719). It did, however, have an impact on the likelihood to switch choices from stimulus color 1 to stimulus color 2 or vice versa. Across participants, a choice switch was more likely to occur following a low value feedback compared with a high value feedback (*t*_(20)_ = 5.10, *p* = 5.4 × 10^−5^).

Feedback in trial n-1 also had an impact on N2pc mean amplitude in trial n. Overall, if a choice was made to the same target in trials n-1 and n (Figure 5A), the N2pc amplitude in trial n was larger following a low value feedback in trial n-1 (Δ_(contra-ipsi)_ = −2.041 ± 0.30 μV), compared with following a high value feedback in trial n-1 (Δ_(contra-ipsi)_ = −1.671 ± 0.33 μV; *F*_(1,20)_ = 4.52, *p* = 0.046; Figure 5B). This was also the case when we did not explicitly control for the choice in trial n (i.e., a choice switch could occur from trial n-1 to trial n, *F*_(1,20)_ = 5.51, *p* = 0.029, data not shown). We were interested in whether feedback had a differential effect on N2pc amplitudes depending on the current state of learning, and therefore separated trials into *during* learning and *after* learning trials. We did not find a significant interaction between the factors feedback and learning in a two-way ANOVA (*F*_(1,20)_ = 0.82, *p* = 0.375). Nevertheless, the difference in N2pc amplitudes following high vs. low value feedback tended to be greater *during* learning than *after* learning (Figure 5C). As previously, this should be treated as suggestive rather than definitive.

Across individual participants, this difference in N2pc amplitude following high vs. low value feedback was significantly correlated with learning performance *during* learning (Figure 5D, left), but not *after* learning (Figure 5D, right). Specifically, the greater the individual difference in N2pc amplitude following high vs. low value feedback *during* learning, the faster the individual learned, i.e., T_Learn_ was smaller (Pearson correlation, *R* = −0.545, *p* = 0.011; Figure 5D, left). However, the difference in N2pc amplitudes following high vs. low value feedback *after* learning was not related to learning performance (Pearson correlation, *R* = 0.058, *p* = 0.803; Figure 5D, right). This difference in correlation coefficients between *during* learning and *after* learning was significant (*z*-test to compare *R*-values, *z* = 2.12, *p* = 0.034). The difference in N2pc amplitude following high vs. low value feedback was not correlated with average RT or the proportion of blocks learned (all *p* > 0.05).

### Feedback Processing Is Increased *during* Learning

Considering the finding that feedback has a differential effect on N2pc amplitudes, and that this effect specifically *during* learning correlates with successful learning behavior, we asked whether feedback processing was affected by learning. We therefore computed the mean amplitude of the FRN as a proxy for negative feedback processing (Miltner et al., 1997). The FRN, computed as the difference between low and high value feedback presentation, was measured at the Fz electrode, since the amplitude difference between low and high value feedback was largest at this electrode. The qualitative and quantitative pattern of results did not change when the FRN was measured at the FCz electrode instead (data not shown). The analysis was done within the 250–325 ms after feedback presentation, since the difference between low and high value was largest in this window (see below) and it generally fell within the range used in the literature (for review see Walsh and Anderson, 2012). We found that processing of feedback (low and high value) was generally increased *during* learning compared with *after* learning (Figure 6A), and the difference between low and high value feedback (FRN) was more pronounced *during* learning compared with *after* learning (*during*: Δ_(lowFB-highFB)_ = −0.791 ± 0.17 μV; *after*: Δ_(lowFB-highFB)_ = −0.426 ± 0.18 μV). The resulting FRN was therefore substantially larger *during* learning as compared with *after* learning (Figure 6B). This was confirmed with a two-way ANOVA that showed a significant main effect of feedback value (*F*_(1,20)_ = 14.70, *p* = 0.001), a significant main effect of learning (*F*_(1,20)_ = 37.18, *p* < 0.001), and a significant interaction between the two parameters (*F*_(1,20)_ = 6.04, *p* = 0.023). Topographical maps of the amplitude difference between low and high value feedback *during* and *after* learning are shown in Figure 6C.

In addition to the change in FRN amplitude with learning, visual inspection of the plots (Figure 6A), revealed a much longer effect of learning that was distinct from the effect of feedback value and the interaction of feedback value and learning. To tease apart these effects and to determine the time range of the effect of learning, we ran a three-way ANOVA with the factors learning (*during* learning, *after* learning), feedback value (high, low), and time window, where we defined 12 50 ms non-overlapping time windows running from 0 ms to 600 ms following feedback onset. We found interactions between the factors learning and time window (*F*_(11,220)_ = 7.13, *p* < 0.001), and feedback value and time window (*F*_(11,220)_ = 3.88, *p* < 001). Follow-up simple main effects across time windows showed that feedback processing *per se* differed with learning in all time windows from 150 ms to 400 ms following feedback onset (F-values between 18.07 and 47.29, all *p* < 0.005, *p-values* in all other time windows >0.05, Bonferroni-Holm multiple comparison corrected). A simple main effect of feedback was only found in the 250–300 ms time window (*F*_(1,20)_ = 16.39, *p* = 0.008, all other *p* > *0*.05, Bonferroni-Holm multiple comparison corrected), confirming the initial FRN analysis above. The previous suggests that feedback processing independent of valence was increased *during* learning in the time window from 150 ms to 400 ms following feedback onset (Figure 6D).

## Discussion

In this study, we implemented a value-based reversal learning task to explore in more detail how attentional target selection and feedback processing is realized in a highly volatile task environment. We measured the N2pc, an EEG component thought to reflect attentional target selection, and the FRN, an EEG component thought to reflect negative feedback processing or prediction error encoding, while participants performed a value-based reversal learning task with probabilistic feedback. Participants were required to frequently adjust their color-based stimulus choice using recent reward feedback. We found that: (i) participants used feedback efficiently to reverse back and forth between the two stimulus choices in accordance with the reversal of their respective values (Figures 1A, 2, 3); (ii) successful learning of the current value-contingency led to a decrease in N2pc amplitudes, which was particularly evident for non-chosen (distractor) stimuli compare with chosen (target) stimuli (Figure 4); (iii) negative feedback in the previous trial was associated with an increase in N2pc mean amplitude, which was selectively correlated with an enhanced learning rate *during* learning (Figure 5); and (iv) FRN amplitudes were increased *during* learning of the current value contingencies, which co-occurred with a more general increase of feedback processing *during* learning (Figure 6).

We live in an environment that is usually much more uncertain and volatile than a classical experimental setting, in which objects or actions need to be continuously evaluated for their relevance to our current goal. We attempted to imitate some of this volatility by employing a value-based reversal learning task, albeit one that is clearly more restrictive than the world outside the laboratory.

To the best of our knowledge this is the first study that investigated learned value-dependent changes of the N2pc amplitude elicited by chosen (i.e., selected) and non-chosen (non-selected) stimuli in a task in which participants were free to select a stimulus for response. Most studies that have investigated the effects of value or reward on attentional stimulus selection in behavior, have used tasks with a predefined, fixed target and distractor-assignment, and in which the trial-by-trial association between the specific stimulus features and reward were in fact irrelevant to solving the task (Della Libera and Chelazzi, 2009; Hickey et al., 2010, 2015; Anderson et al., 2011a, 2013; Itthipuripat et al., 2015; Sawaki et al., 2015; Feldmann-Wüstefeld et al., 2016), often to dissociate reward-related processes from goal-related processes of attentional selection. Or they have used tasks in which value associations of targets or cues were kept constant (Kiss et al., 2009; Raymond and O’Brien, 2009; Krebs et al., 2010; Le Pelley et al., 2013; San Martín et al., 2016), or if they changed, were specifically trained (Navalpakkam et al., 2010). None of these studies allowed insight into how attentional selection changes when the values of target stimuli change unannounced and require repeated adjustment of behavior.

We used probabilistic feedback so that subjects needed to keep track of feedback over multiple trials to determine the current high-value stimulus. Following an unannounced value reversal, participants tended to continue to choose the low value (previously high value) stimulus for response for multiple trials, before switching their choice behavior to the current high value stimulus. RTs were longer *during* learning than *after* learning (Figure 3C), suggesting that participants optimized their attention allocation to stimulus features with learning of the current value contingencies.

Since both stimuli were repeatedly associated with a high and a low value, both were frequently selected for a response. Thus, the dissociation between target and distractor is not as clear on a trial by trial basis as in previous literature (see above). For this reason, it was initially unclear to what extent processing of the chosen (that is, the selected target) stimulus would be enhanced throughout learning and how efficiently the brain could evade attentional capture by the non-chosen (non-selected distractor) stimulus, which always posed a distraction to solving the task quickly and accurately. We initially predicted that as attentional prioritization shifts towards the chosen stimulus with learning, this would concomitantly be reflected by an N2pc increase for the chosen stimulus (Figure 1B, left) and an N2pc decrease for the non-chosen stimulus (Figure 1B, right). Instead we found that N2pc amplitudes decreased with learning in general, which was true on average for both chosen and non-chosen stimuli. However, this decrease in amplitude with learning seemed more apparent for the non-chosen stimulus (Figure 4C). This suggests that the primary effect of learning in this task was a decrease in attention to the non-chosen lateralized stimulus, which potentially indicates suppression (Figure 4Cii). In contrast, the amplitude of the N2pc that reflected processing of the chosen lateralized stimulus did not seem to substantially change with learning (Figure 4Ci). Thus, we find some evidence for our initial hypothesis of how learning affects processing of non-chosen stimuli (Figure 1B, right), but not for our hypothesis of how learning affects processing of chosen stimuli (Figure 1B, left). These results suggest that following a value reversal, when participants needed to actively re-evaluate their current choices, distraction by the non-chosen stimulus was not as effectively evaded as *after* learning, when participants often showed plateau-performance with a high probability of choosing the currently high-valued stimulus (Figures 2, 3).

These results suggest that efficient attention allocation in this highly volatile task design was more likely observed for processing of the non-chosen stimulus in the form of a reduced or suppressed N2pc, and not as an N2pc enhancement of the chosen stimulus. We should note however that although we observed suppression for lateralized non-chosen stimuli, independent of learning, the amplitude elicited in this time interval was still negative, and not positive as has been observed previously (e.g., Hickey et al., 2009; Feldmann-Wüstefeld et al., 2015, 2016; Sawaki et al., 2015). It is therefore difficult to decide whether value learning has led to a reduced capture by the non-chosen (i.e., non-selected) stimulus or an actual suppression, as both interpretations would account for a reduction in N2pc amplitude. Similarly, it is possible that in a less volatile task design in which learning takes place over a much longer time window (e.g., days), an effect of learning on attentional processing would predominantly be observed for the chosen (target) stimulus, as has been shown previously (Clark et al., 2015; Itthipuripat et al., 2017). We should therefore be careful of over-interpreting the absence of a strong effect of learning on N2pc amplitudes of the chosen stimulus in this task, as such an effect could have been revealed with a larger number of *after*-learning trials.

That attention and learning are closely intertwined concepts has been the subject of attentional learning theories for some time (Mackintosh, 1975; Pearce and Hall, 1980; Le Pelley, 2004). The Mackintosh and the Pearce and Hall models of attentional learning predict contradicting relationships between attention and learning. According to Mackintosh (1975), attention is biased towards stimuli that have a higher predictive value, as they are more likely to yield a rewarding outcome (e.g., Mackintosh and Little, 1969; Le Pelley et al., 2013). The Pearce and Hall model on the contrary predicts that unexpected and surprising outcomes that lead to a prediction error are associated with an increase in attention (e.g., Wilson et al., 1992; Anderson et al., 2013). Both theories have received extensive empirical support (Pearce and Mackintosh, 2010) and there have been efforts to reconcile their findings (Holland and Gallagher, 1999; Dayan et al., 2000; Le Pelley, 2004; Hogarth et al., 2010). One possible solution suggests that there is a distinction between two aspects of attention in associative learning: attention that is concerned with action, and attention that is concerned with learning. It suggests that one should attend to the most reward-predicting stimuli or features when making a choice, but should attend to the most uncertain stimuli or features when learning from prediction errors (Holland and Gallagher, 1999; Dayan et al., 2000; Hogarth et al., 2010; Gottlieb, 2012).

Even though our task was not designed to address this debate explicitly, we found evidence that in a highly volatile environment that encourages continuous learning, attention is increased during periods of uncertainty in line with Pearce and Hall (1980). In particular, we tested whether feedback itself could influence the allocation of attention to target stimuli and found that low-value feedback was followed by a larger N2pc amplitude on the next trial than was high-value feedback (Figure 5B), when the only difference between two consecutive trials was the feedback received after the first trial (Figure 5A). Importantly, only *during* learning did this difference in N2pc amplitude following low vs. high value feedback correlate with individual participants’ learning rates—participants in which the difference in feedback-dependent N2pc amplitude was particularly large learned faster (Figure 5D, left). This suggests that *during* learning, when participants needed to actively reevaluate their stimulus choices and relearn the current value contingency, allocating more attention to the choice stimulus after experiencing a negative outcome compared with a positive outcome, led to faster and more successful adjustment of behavior according to the current value contingency. In addition, this suggests that in a highly volatile task environment that requires continuous learning and value updating, with sources of uncertainty found in the inherent reward probability distributions (70%–30%) and in the sudden value reversals (Yu and Dayan, 2005; Payzan-LeNestour and Bossaerts, 2011), participants allocate more attention to an uncertain compared with a more certain choice stimulus, in line with Pearce and Hall (1980). It is possible that in a less volatile environment in which participants have much longer periods of consistent choices, i.e., periods in which participants know the current value contingency with high certainty and learning presumably no longer takes place, we could have observed an increase in attention during target selection, which would be in line with Mackintosh (1975). Although we are using the N2pc amplitude as a proxy for selective attention, which may limit the implications to be drawn, our results are consistent with the idea that when tasks demand states of active learning, attention is increased following uncertain choice outcomes or events, and this correlates with enhanced learning performance.

Another prominent EEG component that has been shown to change during reversal learning is the FRN (e.g., Chase et al., 2011; von Borries et al., 2013; Donaldson et al., 2016). The FRN has been thought to encode negative feedback, prediction error signals, outcome valence and behavioral adjustment (Holroyd and Coles, 2002; Cohen and Ranganath, 2007; Bellebaum and Daum, 2008; Chase et al., 2011; Walsh and Anderson, 2011; von Borries et al., 2013; Donaldson et al., 2016). Using a probabilistic reversal learning paradigm similar to ours, Chase et al. (2011) have shown that the FRN amplitude scales with a negative prediction error signal obtained with a reinforcement learning model, whereby the FRN amplitude was largest following a reversal and diminished as a behavioral adjustment approached. Recent evidence using a reversal learning task, in which positive as well as negative outcomes could signal a need for behavioral adjustment and could be equally unexpected, suggests that the FRN may be more related to outcome valence (positive vs. negative) than to expectancy or behavioral adjustment (von Borries et al., 2013; Donaldson et al., 2016). We computed the FRN as the difference wave between the presentation of low-value and high-value feedback, and then compared the difference *during* learning with *after* learning (Figure 6B). We found that the FRN was substantially larger *during* learning than *after* learning. Although we should be careful of over-interpreting, since our task confounds the accumulation of negative outcomes with the need for behavioral adjustment, these results suggest that low-value as well as high-value feedback are processed differently during periods of uncertainty (*during* learning) when stimulus-value associations need to be updated (Figure 6A), which cannot be solely explained by differences in outcome valence.

In addition to the effect of learning on the FRN amplitudes, we also observed a more general and longer lasting effect of learning on feedback processing that was independent of feedback valence (Figure 6D). Feedback processing differed between 150 ms and 400 ms following feedback onset d*uring* learning of current value contingencies compared with *after* learning, which could indicate an increase in feedback processing *per se*. The FRN is thought to originate in anterior cingulate cortex (ACC; Hickey et al., 2010), and this prolonged window of differential feedback processing matches the time-resolved activity level of ACC during reward presentation observed previously (Hickey et al., 2010). In our task, enhanced ACC activity, during periods in which participants experience increased levels of uncertainty that potentially require a behavioral adjustment, could potentially reflect the necessity of increased levels of cognitive control (Shenhav et al., 2013, 2016), or increased activity related to the decision of moving from a state of exploitation to a state of exploration (Kolling et al., 2016). Together with increased attention *during* learning and following negative feedback, these signals may be part of the underlying neural network that drives behavioral adjustment during periods of increased uncertainty that concludes in the switch of stimulus choice.

Overall, we found evidence that during periods of active behavioral adjustment in a changing and volatile task environment, feedback processing of recent choices and attentional processing is amplified and co-occurs with increases in attentional allocation following low-value feedback compared with high-value feedback that possibly promotes increased learning speed. Following successful learning of current value contingencies, during periods of stable behavior, attentional allocation then becomes potentially more efficient by suppressing non-relevant distractor processing. These results provide insight into how changes in attentional prioritization and feedback processing may support flexible and repeated behavioral adjustments in humans.

## Author Contributions

MO, MRW, TW and AS designed the experiment. MO collected the data, performed the analyses and drafted the manuscript. All authors edited the manuscript.

## Conflict of Interest Statement

The authors declare that the research was conducted in the absence of any commercial or financial relationships that could be construed as a potential conflict of interest.

## Footnotes

^1^http://www.ru.nl/fcdonders/fieldtrip/

## References

[B1] Anderson B. A. (2013). A value-driven mechanism of attentional selection. J. Vis. 13:7. 10.1167/13.3.7 23589803 PMC3630531

[B2] Anderson B. A. Laurent P. A. Yantis S. (2011a). Learned value magnifies salience-based attentional capture. PLoS One 6:e27926. 10.1371/journal.pone.0027926 22132170 PMC3221688

[B3] Anderson B. A. Laurent P. A. Yantis S. (2011b). Value-driven attentional capture. Proc. Natl. Acad. Sci. U S A 108, 10367–10371. 10.1073/pnas.1104047108 21646524 PMC3121816

[B4] Anderson B. A. Laurent P. A. Yantis S. (2013). Reward predictions bias attentional selection. Front. Hum. Neurosci. 7:262. 10.3389/fnhum.2013.00262 23781185 PMC3678100

[B5] Anderson B. A. Laurent P. A. Yantis S. (2014). Value-driven attentional priority signals in human basal ganglia and visual cortex. Brain Res. 1587, 88–96. 10.1016/j.brainres.2014.08.062 25171805 PMC4253668

[B6] Awh E. Belopolsky A. V. Theeuwes J. (2012). Top-down versus bottom-up attentional control: a failed theoretical dichotomy. Trends Cogn. Sci. 16, 437–443. 10.1016/j.tics.2012.06.010 22795563 PMC3426354

[B7] Bellebaum C. Daum I. (2008). Learning-related changes in reward expectancy are reflected in the feedback-related negativity. Eur. J. Neurosci. 27, 1823–1835. 10.1111/j.1460-9568.2008.06138.x 18380674

[B8] Bucker B. Theeuwes J. (2017). Pavlovian reward learning underlies value driven attentional capture. Atten. Percept. Psychophys. 79, 415–428. 10.3758/s13414-016-1241-1 27905069 PMC5306301

[B9] Chase H. W. Swainson R. Durham L. Benham L. Cools R. (2011). Feedback-related negativity codes prediction error but not behavioral adjustment during probabilistic reversal learning. J. Cogn. Neurosci. 23, 936–946. 10.1162/jocn.2010.21456 20146610

[B10] Clark K. Appelbaum L. G. van den Berg B. Mitroff S. R. Woldorff M. G. (2015). Improvement in visual search with practice: mapping learning-related changes in neurocognitive stages of processing. J. Neurosci. 35, 5351–5359. 10.1523/JNEUROSCI.1152-14.2015 25834059 PMC4381005

[B11] Cohen M. X. Elger C. E. Ranganath C. (2007). Reward expectation modulates feedback-related negativity and EEG spectra. Neuroimage 35, 968–978. 10.1016/j.neuroimage.2006.11.056 17257860 PMC1868547

[B12] Cohen M. X. Ranganath C. (2007). Reinforcement learning signals predict future decisions. J. Neurosci. 27, 371–378. 10.1523/JNEUROSCI.4421-06.2007 17215398 PMC6672075

[B13] Cools R. Clark L. Owen A. M. Robbins T. W. (2002). Defining the neural mechanisms of probabilistic reversal learning using event-related functional magnetic resonance imaging. J. Neurosci. 22, 4563–4567. 12040063 10.1523/JNEUROSCI.22-11-04563.2002 PMC6758810

[B14] Corbetta M. Shulman G. L. (2002). Control of goal-directed and stimulus-driven attention in the brain. Nat. Rev. Neurosci. 3, 215–229. 10.1038/nrn755 11994752

[B15] Dayan P. Kakade S. Montague P. R. (2000). Learning and selective attention. Nat. Neurosci. 3, 1218–1223. 10.1038/81504 11127841

[B16] Della Libera C. Chelazzi L. (2006). Visual selective attention and the effects of monetary rewards. Psychol. Sci. 17, 222–227. 10.1111/j.1467-9280.2006.01689.x 16507062

[B17] Della Libera C. Chelazzi L. (2009). Learning to attend and to ignore is a matter of gains and losses. Psychol. Sci. 20, 778–784. 10.1111/j.1467-9280.2009.02360.x 19422618

[B18] Della Libera C. Perlato A. Chelazzi L. (2011). Dissociable effects of reward on attentional learning: from passive associations to active monitoring. PLoS One 6:e19460. 10.1371/journal.pone.0019460 21559388 PMC3084870

[B19] Donaldson K. R. Ait Oumeziane B. Hélie S. Foti D. (2016). The temporal dynamics of reversal learning: P3 amplitude predicts valence-specific behavioral adjustment. Physiol. Behav. 161, 24–32. 10.1016/j.physbeh.2016.03.034 27059320 PMC5426362

[B20] Eimer M. (2014). The neural basis of attentional control in visual search. Trends Cogn. Sci. 18, 526–535. 10.1016/j.tics.2014.05.005 24930047

[B21] Eimer M. Grubert A. (2014). Spatial attention can be allocated rapidly and in parallel to new visual objects. Curr. Biol. 24, 193–198. 10.1016/j.cub.2013.12.001 24412208

[B22] Feldmann-Wüstefeld T. Brandhofer R. Schubö A. (2016). Rewarded visual items capture attention only in heterogeneous contexts. Psychophysiology 53, 1063–1073. 10.1111/psyp.12641 26997364

[B23] Feldmann-Wüstefeld T. Schubö A. (2013). Context homogeneity facilitates both distractor inhibition and target enhancement. J. Vis. 13:11. 10.1167/13.3.11 23650629

[B24] Feldmann-Wüstefeld T. Uengoer M. Schubö A. (2015). You see what you have learned. Psychophysiology 52, 1483–1497. 10.1111/psyp.12514 26338030

[B25] Gottlieb J. (2012). Attention, learning, and the value of information. Neuron 76, 281–295. 10.1016/j.neuron.2012.09.034 23083732 PMC3479649

[B26] Hajcak G. Moser J. S. Holroyd C. B. Simons R. F. (2006). The feedback-related negativity reflects the binary evaluation of good versus bad outcomes. Biol. Psychol. 71, 148–154. 10.1016/j.biopsycho.2005.04.001 16005561

[B27] Hickey C. Chelazzi L. Theeuwes J. (2010). Reward changes salience in human vision via the anterior cingulate. J. Neurosci. 30, 11096–11103. 10.1523/JNEUROSCI.1026-10.2010 20720117 PMC6633486

[B28] Hickey C. Chelazzi L. Theeuwes J. (2011). Reward has a residual impact on target selection in visual search, but not on the suppression of distractors. Vis. Cogn. 19, 117–128. 10.1080/13506285.2010.503946

[B29] Hickey C. Di Lollo V. McDonald J. J. (2009). Electrophysiological indices of target and distractor processing in visual search. J. Cogn. Neurosci. 21, 760–775. 10.1162/jocn.2009.21039 18564048

[B30] Hickey C. Kaiser D. Peelen M. V. (2015). Reward guides attention to object categories in real-world scenes. J. Exp. Psychol. Gen. 144, 264–273. 10.1037/a0038627 25559653

[B31] Hogarth L. Dickinson A. Duka T. (2010). “Selective attention to conditioned stimuli in human discrimination learning: untangling the effects of outcome prediction, valence, arousal, and uncertainty,” in Attention and Associative Learning. From Brain to Behavior, eds Mitchell C. J. Le Pelley M. E. (Oxford: Oxford University Press), 71–97.

[B32] Holland P. C. Gallagher M. (1999). Amygdala circuitry in attentional and representational processes. Trends Cogn. Sci. 3, 65–73. 10.1016/s1364-6613(98)01271-6 10234229

[B33] Holroyd C. Coles M. (2002). The neural basis of human error processing: reinforcement learning, dopamine, and the error-related negativity. Psychol. Rev. 109, 679–709. 10.1037/0033-295X.109.4.679 12374324

[B34] Hopf J. M. Luck S. J. Boelmans K. Schoenfeld M. A. Boehler C. N. Rieger J.. (2006). The neural site of attention matches the spatial scale of perception. J. Neurosci. 26, 3532–3540. 10.1523/JNEUROSCI.4510-05.2006 16571761 PMC6673866

[B35] Hopf J. M. Luck S. J. Girelli M. Hagner T. Mangun G. R. Scheich H.. (2000). Neural sources of focused attention in visual search. Cereb. Cortex 10, 1233–1241. 10.1093/cercor/10.12.1233 11073872

[B36] Irons J. L. Leber A. B. (2016). Choosing attentional control settings in a dynamically changing environment. Atten. Percept. Psychophys. 78, 2031–2048. 10.3758/s13414-016-1125-4 27188652

[B37] Itthipuripat S. Cha K. Byers A. Serences J. T. (2017). Two different mechanisms support selective attention at different phases of training. PLoS Biol. 15:e2001724. 10.1371/journal.pbio.2001724 28654635 PMC5486967

[B38] Itthipuripat S. Cha K. Rangsipat N. Serences J. T. (2015). Value-based attentional capture influences context-dependent decision-making. J. Neurophysiol. 114, 560–569. 10.1152/jn.00343.2015 25995350 PMC4509404

[B39] Kastner S. Ungerleider L. G. (2000). Mechanisms of visual attention in the human cortex. Annu. Rev. Neurosci. 23, 315–341. 10.1146/annurev.neuro.23.1.315 10845067

[B40] Kiss M. Driver J. Eimer M. (2009). Reward priority of visual target singletons modulates ERP signatures of attentional selection. Psychol. Sci. 20, 245–251. 10.1111/j.1467-9280.2009.02281.x 19175756 PMC2645377

[B93] Kiss M. Van Velzen J. Eimer M. (2008). The N2pc component and its links to attention shifts and spatially selective visual processing. Psychophysiology 45, 240–249. 10.1111/j.1469-8986.2007.00611.x 17971061 PMC2248220

[B41] Kolling N. Wittmann M. K. Behrens T. E. J. Boorman E. D. Mars R. B. Rushworth M. F. S. (2016). Value, search, persistence and model updating in anterior cingulate cortex. Nat. Neurosci. 19, 1280–1285. 10.1038/nn.4382 27669988 PMC7116891

[B42] Krebs R. M. Boehler C. N. Woldorff M. G. (2010). The influence of reward associations on conflict processing in the Stroop task. Cognition 117, 341–347. 10.1016/j.cognition.2010.08.018 20864094 PMC2967668

[B43] Le Pelley M. E. (2004). The role of associative history in models of associative learning: a selective review and a hybrid model. Q. J. Exp. Psychol. B 57, 193–243. 10.1080/02724990344000141 15204108

[B44] Le Pelley M. E. Vadillo M. Luque D. (2013). Learned predictiveness influences rapid attentional capture: evidence from the dot probe task. J. Exp. Psychol. Learn. Mem. Cogn. 39, 1888–1900. 10.1037/a0033700 23855549

[B91] Luck S. J. Hillyard S. A. (1994a). Electrophysiological correlates of feature analysis during visual search. Psychophysiology 31, 291–308. 10.1111/j.1469-8986.1994.tb02218.x 8008793

[B92] Luck S. J. Hillyard S. A. (1994b). Spatial filtering during visual search: evidence from human electrophysiology. J. Exp. Psychol. Hum. Percept. Perform. 20, 1000–1014. 10.1037/0096-1523.20.5.1000 7964526

[B46] Mackintosh N. J. (1975). A theory of attention: variations in the associability of stimuli with reinforcement. Psychol. Rev. 82, 276–298. 10.1037/h0076778

[B47] Mackintosh N. J. Little L. (1969). Intradimensional and extradimensional shift learning by pigeons. Psychon. Sci. 14, 5–6. 10.3758/bf03336395

[B48] Miltner W. H. R. Braun C. H. Coles M. G. H. (1997). Event-related brain potentials following incorrect feedback in a time-estimation task: evidence for a “generic” neural system for error detection. J. Cogn. Neurosci. 9, 788–798. 10.1162/jocn.1997.9.6.788 23964600

[B90] Munneke J. Hoppenbrouwers S. S. Theeuwes J. (2015). Reward can modulate attentional capture, independent of top-down set. Atten. Percept. Psychophys. 77, 2540–2548. 10.3758/s13414-015-0958-6 26178858 PMC4644218

[B49] Navalpakkam V. Koch C. Rangel A. Perona P. (2010). Optimal reward harvesting in complex perceptual environments. Proc. Natl. Acad. Sci. U S A 107, 5232–5237. 10.1073/pnas.0911972107 20194768 PMC2841865

[B50] Payzan-LeNestour E. Bossaerts P. (2011). Risk, unexpected uncertainty and estimation uncertainty: bayesian learning in unstable settings. PLoS Comput. Biol. 7:e1001048. 10.1371/journal.pcbi.1001048 21283774 PMC3024253

[B51] Pearce J. Hall G. (1980). A model for Pavlovian learning: variation in the effectiveness of conditioned but not unconditioned stimuli. Psychol. Rev. 87, 532–552. 10.1037/0033-295x.87.6.532 7443916

[B52] Pearce J. Mackintosh N. J. (2010). “Two theories of attention: a review and possible integration,” in Attention and Associative Learning. From Brain to Behavior, eds Mitchell C. J. Le Pelley M. E. (Oxford: Oxford University Press), 11–39.

[B53] Posner M. Petersen S. E. (1990). The attention system of the human brain. Annu. Rev. Neurosci. 13, 25–42. 10.1146/annurev.ne.13.030190.000325 2183676

[B54] Raymond J. E. O’Brien J. L. (2009). Selective visual attention and motivation: the consequences of value learning in an attentional blink task. Psychol. Sci. 20, 981–989. 10.1111/j.1467-9280.2009.02391.x 19549080

[B55] Sali A. W. Anderson B. A. Yantis S. (2014). The role of reward prediction in the control of attention. J. Exp. Psychol. Hum. Percept. Perform. 40, 1654–1664. 10.1037/a0037267 24955700 PMC4313538

[B56] San Martín R. Appelbaum L. G. Huettel S. A. Woldorff M. G. (2016). Cortical brain activity reflecting attentional biasing toward reward-predicting cues covaries with economic decision-making performance. Cereb. Cortex 26, 1–11. 10.1093/cercor/bhu160 25139941 PMC4677969

[B57] Sawaki R. Luck S. J. Raymond J. E. (2015). How attention changes in response to incentives. J. Cogn. Neurosci. 27, 2229–2239. 10.1162/jocn_a_00847 26151604 PMC4589447

[B58] Shenhav A. Botvinick M. M. Cohen J. D. (2013). The expected value of control: an integrative theory of anterior cingulate cortex function. Neuron 79, 217–240. 10.1016/j.neuron.2013.07.007 23889930 PMC3767969

[B59] Shenhav A. Cohen J. D. Botvinick M. M. (2016). Dorsal anterior cingulate cortex and the value of control. Nat. Neurosci. 19, 1286–1291. 10.1038/nn.4384 27669989

[B60] Smith A. C. Brown E. N. (2003). Estimating a state-space model from point process observations. Neural Comput. 15, 965–991. 10.1162/089976603765202622 12803953

[B61] Smith A. C. Frank L. M. Wirth S. Yanike M. Hu D. Kubota Y.. (2004). Dynamic analysis of learning in behavioral experiments. J. Neurosci. 24, 447–461. 10.1523/JNEUROSCI.2908-03.2004 14724243 PMC6729979

[B62] Steiger J. H. (1980). Tests for comparing elements of a correlation matrix. Psychol. Bull. 87, 245–251. 10.1037/0033-2909.87.2.245

[B63] von Borries A. K. L. Verkes R. J. Bulten B. H. Cools R. de Bruijn E. R. A. (2013). Feedback-related negativity codes outcome valence, but not outcome expectancy, during reversal learning. Cogn. Affect. Behav. Neurosci. 13, 737–746. 10.3758/s13415-013-0150-1 24146314

[B64] Walsh M. M. Anderson J. R. (2011). Modulation of the feedback-related negativity by instruction and experience. Proc. Natl. Acad. Sci. U S A 208, 19048–19053. 10.1073/pnas.1117189108 22065792 PMC3223452

[B65] Walsh M. M. Anderson J. R. (2012). Learning from experience: event-related potential correlates of reward processing, neural adaptation, and behavioral choice. Neurosci. Biobehav. Rev. 36, 1870–1884. 10.1016/j.neubiorev.2012.05.008 22683741 PMC3432149

[B66] Wilson P. N. Boumphrey P. Pearce J. M. (1992). Restoration of the orienting response to a light by a change in its predictive accuracy. J. Exp. Psychol. 44, 17–36.

[B67] Womelsdorf T. Everling S. (2015). Long-range attention networks: circuit motifs underlying endogenously controlled stimulus selection. Trends Neurosci. 38, 682–700. 10.1016/j.tins.2015.08.009 26549883

[B94] Woodman G. F. Luck S. J. (1999). Electrophysiological measurement of rapid shifts of attention during visual search. Nature 400, 867–869. 10.1038/23698 10476964

[B68] Woodman G. F. Luck S. J. (2003). Serial deployment of attention during visual search. J. Exp. Psychol. Hum. Percept. Perform. 29, 121–138. 10.1037/0096-1523.29.1.121 12669752

[B69] Yu A. J. Dayan P. (2005). Uncertainty, neuromodulation, and attention. Neuron 46, 681–692. 10.1016/j.neuron.2005.04.026 15944135

